# Establishment of a Markerless Mutation Delivery System in *Bacillus subtilis* Stimulated by a Double-Strand Break in the Chromosome

**DOI:** 10.1371/journal.pone.0081370

**Published:** 2013-11-25

**Authors:** Ting Shi, Guanglu Wang, Zhiwen Wang, Jing Fu, Tao Chen, Xueming Zhao

**Affiliations:** 1 Department of Biochemical Engineering, School of Chemical Engineering and Technology, Tianjin University, Tianjin, People’s Republic of China; 2 Key Laboratory of Systems Bioengineering, Ministry of Education, Tianjin University, Tianjin, People’s Republic of China; 3 Collaborative Innovation Center of Chemical Science and Engineering, Tianjin, People’s Republic of China; 4 Edinburgh-Tianjin Joint Research Centre for Systems Biology and Synthetic Biology, Tianjin University, Tianjin, People’s Republic of China; Loyola University Medical Center, United States of America

## Abstract

*Bacillus subtilis* has been a model for gram-positive bacteria and it has long been exploited for industrial and biotechnological applications. However, the availability of facile genetic tools for physiological analysis has generally lagged substantially behind traditional genetic models such as *Escherichia coli* and *Saccharomyces cerevisiae*. In this work, we have developed an efficient, precise and scarless method for rapid multiple genetic modifications without altering the chromosome of *B. subtilis*. This method employs *upp* gene as a counter-selectable marker, double-strand break (DSB) repair caused by exogenous endonuclease I-S*ce*I and *comK* overexpression for fast preparation of competent cell. Foreign dsDNA can be simply and efficiently integrated into the chromosome by double-crossover homologous recombination. The DSB repair is a potent inducement for stimulating the second intramolecular homologous recombination, which not only enhances the frequency of resolution by one to two orders of magnitude, but also selects for the resolved product. This method has been successfully and reiteratively used in *B. subtilis* to deliver point mutations, to generate in-frame deletions, and to construct large-scale deletions. Experimental results proved that it allowed repeated use of the selectable marker gene for multiple modifications and could be a useful technique for *B. subtilis*.

## Introduction

*Bacillus subtilis* has been applied as a model system for researches on the aspects of biochemistry, genetics and physiology of Gram-positive bacteria, and has long been used as an important cell factory for industrial applications [1]. In recent years, with the rapid development of post-genomic studies in *B. subtilis* such as genome reduction engineering [2], inverse metabolic engineering [3], [4] and synthetic biology [5], it is beneficial to create a simple method to enable multiple genetic modifications for exploration of the molecular mechanisms conveniently. Three essentials including simple and efficient procedure for transformation, suitable counter-selectable marker and high marker recycling efficiency play crucial roles in the markerless genetic modification system.

Transformation efficiency is one of the decisive factors for genetic manipulation. It is desirable to develop an approach for fast preparation of competent cells with high transformation efficiency. As we known, natural transformation is a programmed mechanism characterized by binding of free double-stranded (ds) DNA from the environment to the cell pole in rod-shaped bacteria that is widely used for chromosomal genetic manipulation [6]. Programmed competence coordinates the expression of proteins involved in DNA uptake and translocation with expression of some proteins of the recombination machinery. One of the early competence induced genes encodes the master regulator (ComK) that subsequently regulates the expression of the “late” genes to drive expression a set of genes in wild type cells that are necessary for building up the DNA uptake apparatus and recombination [7]–[10]. Overexpression of *comK* gene or deletion of *rok* gene (encoding the negative regulator of *comK*) is a valuable method for improvement of natural transformation efficiency and has been applied in *B*. *subtilis* [11]–[13]. Induction of *comK* combined with positive auto-stimulation of native *comK* is sufficient for the transcriptional activation of the late competence genes, which results in an increased percentage of competent cells in the population [14] and leads to establishment of the competent state when it enters the stationary growth phase [15].

The effectiveness of counter-selectable marker is correlated with counter-selection efficiency and the practicability of markerless mutation delivery system. A number of systems for marker recycling in *B. subtilis* have been reported based on the following counter-selectable marker: *mazF* [16]–[18], *ysbC* [19], *blaI* [20], *lacI* [21], *hewl* [22] and *upp* [23]. Besides, the site-specific recombinases system such as *Cre/lox* can also be used to delete the selectable marker for counter-selection. However, it cannot be applied in allele replacement and the “scar” sequence left at the modification locus might be unwanted in some cases [22], [24]. Applications of *mazF* and *ysbC* are difficult to be maintained in *E. coli*, whereas *blaI* and *lacI* require the corresponding auxotrophic host strains. The *hewl* gene is substantiated to be active against *Bacillus* species recently and can be used as a powerful marker for counter-selection [22]. The *upp* gene has been widely used in markerless modifications, such as gene disruption, point mutation, and large-scale deletion [23], [25]–[28]. Although it is feasible as a counter-selectable marker, the proportion of cells which undergo pop-out is expected to be of the order of 10^−6^ [23] and actually needs to be enhanced to facilitate the application of markerless system.

The I-S*ce*I based mutagenesis system for unmarked genetic manipulation has been broadly employed in several microorganisms to promote the efficiency of resolution [29]–[33]. The exogenous endonuclease I-S*ce*I, as a counter-selection tool, causes a unique double-strand break (DSB) with a 4-base 3′ hydroxyl overhang [34] at the I-S*ce*I recognition site in the genome. This broken chromosomal ends are then subjected to RecA-mediated DSB repair, which is a potent inducement to stimulate homologous recombination within the regions flanked by the broken ends. Fortunately, genome of *B. subtilis* 168 is free of the 18 bp I-S*ce*I recognition sequence, which was affirmed when no genome DNA degradation was detected after treatment with I-S*ce*I [35].

In the present work, we introduce a simple and general strategy to create markerless genetic manipulations in *B. subtilis*. This procedure, based on competence fast preparation and repair activities of the cell, utilizes *upp* as counter-selectable marker in concert with DSB stimulation repair to construct various mutations efficiently. It simplifies experimental procedure and shortens the processing time to generate modification without altering the chromosome in any other condition. Experimental results demonstrate that point mutation, in-frame deletion and large-scale deletion can be readily made with this novel approach. It is simple, fast and of high efficiency in contrast to routine method, promoting it as an attractive and useful tool for genome editing.

## Materials and Methods

### Bacterial Strains, Plasmids, Primers and Growth Conditions

Table 1 lists the bacterial strains and plasmids used in this study. The information of primers is summarized (Table S1). All *B. subtilis* mutant strains were derived from the *B. subtilis* Marburg 168 (*trpC2*). Strains were grown at 37°C in Luria–Bertani (LB) liquid medium or M9 liquid medium [36] for genetics and physiology characterization and minimal medium (MM) [23] for counter-selection of the 5-fluorouracil (5FU) resistance colonies. Solid media was obtained by adding 1.5% agar to the liquid media. When required, antibiotics were added to the growth media at the following concentrations: 100 µg/ml ampicillin for *E. coli* selection; 100 µg/ml spectinomycin, 5 µg/ml kanamycin, 5 µg/ml chloramphenicol, and 1 µg/ml erythromycin for *B. subtilis* selection; 5FU was purchased from Sigma-Aldrich Corporation (Sigma-Aldrich, St Louis, MO, USA) and prepared as a stock solution of 100 mM in dimethyl sulfoxide (DMSO). Colonies that popped out the *upp*-cassette were selected on MM plate containing 10 µM 5FU.

**Table 1:** Bacterial strains and plasmids used in this study.

Strain or plasmid	Relevant description(s)^a^	Reference or source
Strains		
*E. coli* DH5α	Standard cloning strain	Invitrogen
*B. subtilis* 168	Wild-type strain, *trpC2*	Laboratory stock
*B. subtilis* BUK	*B. subtilis* 168 derivate, Δ*upp*::*P_ara_*-*comK*	This study
*B. subtilis* BUK-1C	*B. subtilis* 168 derivate, Δ*upp*::*P_ara_*-*comK*, Δ*ccpN*::*upp*-cassette	This study
*B. subtilis* BUK-1	*B. subtilis* 168 derivate, Δ*upp*::*P_ara_*-*comK*, *ccpN* (Ala 130 to Ser)	This study
*B. subtilis* BUK-2C	*B. subtilis* 168 derivate, Δ*upp*::*P_ara_*-*comK*, Δ*ccpN*::*upp*-cassette	This study
*B. subtilis* BUK-2	*B. subtilis* 168 derivate, Δ*upp*::*P_ara_*-*comK*, Δ*ccpN*	This study
*B. subtilis* BUK-3C	*B. subtilis* 168 derivate, Δ*upp*::*P_ara_*-*comK*, Δ*ileS*::*upp*-cassette-ileS*	This study
*B. subtilis* BUK-3	*B. subtilis* 168 derivate, Δ*upp*::*P_ara_*-*comK*, *ileS* (Ala 467 to Thr)	This study
*B. subtilis* BUKΔ20.5 kb	*B. subtilis* 168 derivate, Δ*ydcL-yddN*	This study
*B. subtilis* BUKΔ75.9 kb	*B. subtilis* 168 derivate, Δ*mutL-pksR*	This study
Plasmids^b^		
pAX01	*B. subtilis* integration vector, *P_xylA_*, Amp^R^, Em^R^	BGSC^a^
pDK	*B. subtilis* integration vector, Km^R^	BGSC^a^
pUC18	*E. coli* cloning vector, *oriT*, Amp^R^	Laboratory stock
pTKRED	*E. coli* cloning vector, I-S*ce*I, Spc^R^	Laboratory stock
pE194	*Bacillus*/*Staphylococcus* cloning vector, Em^R^	Laboratory stock
pSS	Modular vector carrying *upp*-cassette, Amp^R^, Cm^R^	This study
pST	*B. subtilis* integration vector, *P_ara_*-*comK*, Amp^R^	This study
pEB	*B. subtilis*-*E. coli* shuttle vector, Amp^R^, Em^R^	This study
pEBS	*B. subtilis*-*E. coli* shuttle vector, *P_xylA_*-I-S*ce*I, Amp^R^, Em^R^	This study
pEBS-*cop*1	pEBS carrying a point mutation in *cop*-1 site, Em^R^	This study
pSS-*ileS*	*B. subtilis* integration vector, Cm^R^	This study

a *Bacillus* Genetic Stock Center.

b Antibiotic resistance genes in plasmids were abbreviated as follows: Amp^R^, ampicillin resistance; Cm^R^, chloramphenicol resistance; Em^R^, erythromycin resistance; Spc^R^, spectinomycin resistance; Km^R^, kanamycin resistance.

### Construction of the Modular Cassette

**Figure 1:**
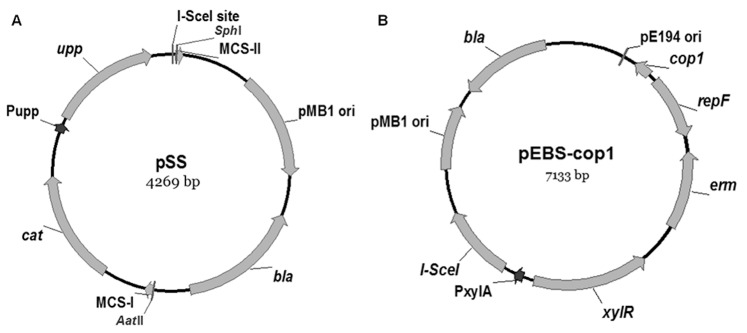
Plasmids for genetic modification in *B. subtilis*. (A) A modular cassette was cloned into pUC18 to generate pSS, the modular cassette contained chloramphenicol resistance gene (*cat*), UPRTase encoding gene (*upp*) and the recognition site of I-S*ce*I endonuclease at 3′ end of *upp* gene. (B) The xylose-inducible I-S*ce*I cassette was cloned into pEB, with a temperature-sensitive replicon (*repF*) and erythromycin resistance gene (*erm*).

The *upp* gene, coding uracil phosphoribosyl-transferase, was fused to a chloramphenicol resistance gene (*cat*) and the recognition site of I-S*ce*I endonuclease to create an “*upp*-cassette” for marker recycling. It was assembled from different templates by the following steps. The *cat* gene was PCR-generated from vector pC194 using primer pair pSS-P1/pSS-P2, with a multiple clone site (MCS) introduced by the forward primer pSS-P1. In parallel, the *upp* gene with its 5′ regulatory region and 3′ transcription terminator was amplified from *B. subtilis* 168 using primer pair pSS-P3/pSS-P4, with an 18 bp oligonucleotide of I-S*ce*I endonuclease recognition site introduced by the reverse primer pSS-P4. Then both PCR fragments were seamlessly joined by splice overlap extension PCR (SOE-PCR) using primer pair pSS-P1/pSS-P4 to generate the *upp*-cassette, where the two genes were oriented in the same direction. Subsequently, the cassette was digested with *Aat*II and *Sph*I and cloned into the same sites of pUC18 to yield modular vector pSS (Figure 1A). The 2 kb selection-eviction *upp*-cassette, was amplified from pSS using appropriate primers (Table S1).

### Construction of pEBS-*cop*1

To stimulate intramolecular recombination by DSB repair in *B. subtilis*, we constructed a temperature-sensitive shuttle vector pEBS-*cop*1 (Figure 1B). A region including a temperature-sensitive replication origin and the erythromycin resistance gene (*erm*) was amplified from pE194 using primer pair pEBS-P1/pEBS-P2 and cloned between *Hind*III and *Pst*I sites of pUC18, forming an *E. coli*-*B. subtilis* shuttle vector pEB. The I-S*ce*I gene was amplified from pTKRED using primer pair pEBS-P3/pEBS-P4, and inserted into *Bam*HI and *Nco*I sites of pAX01 to generate pAX01-S*ce*I. Subsequently, a xylose-inducible I-S*ce*I expression cassette was PCR-generated using pEBS-P5/pEBS-P6 as primers and pAX01-S*ce*I as template, and inserted between *Kpn*I and *Pst*I sites of pEB to yield pEBS with a low copy number of about 10 at 30°C [37].

To acquire a high copy number, site-directed mutagenesis was carried out to mutate the promoter of *cop* gene in pEBS by using Phusion TM site-directed mutagenesis kit (New England BioLabs, Ipswich, MA) according to the manufacturer’s instructions. The pEBS-*cop*1 was obtained which contained a C•G→G•C nucleotide transversion in the *cop* promoter region, with a high copy number of about 200 at 30°C [37]. It could be stably maintained under the selection pressure in *B. subtilis* cells at 30°C, but the copy number dramatically decreased when cells were grown at temperatures above 37°C and no growth occurred at 50°C in the presence of selective antibiotic.

### Construction of pST

**Figure 2:**
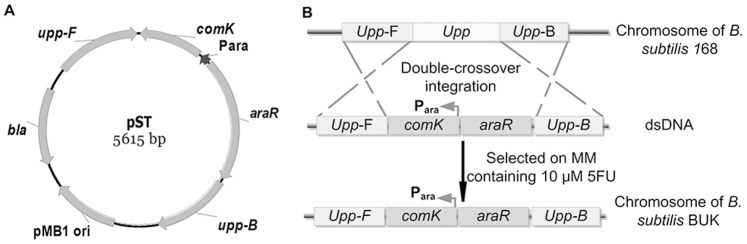
Construction of host strain B. subtilis BUK. (A) Two homologous arms of *upp* gene (*Upp*-F and *Upp*-B) were cloned into pUC18, and the arabinose-inducible *comK* expression cassette was fused downstream of the front homologous arm to construct vector pST. (B) The sequence between two homologous arms of *upp* gene (*Upp*-F and *Upp*-B), was replaced by *comK* cassette via a double-crossover recombination. *Crossed lines* indicated double-crossover recombinant events. *Upp*-F and *Upp*-B, upstream and downstream fragment of *upp* gene, respectively; *comK*, the master regulator ComK encoding gene for *B. subtilis* competence development; *P_ara_*, arabinose-inducible promoter of *ara* operon; *araR*, arabinose operon transcriptional repressor of *B. subtilis*.

Plasmid pST was constructed for *upp* deletion and *comK* expression cassette insertion at the *upp* locus on *B. subtilis* 168 chromosome. The *upp* upstream region (787 bp) was amplified using primer pair pST-P1/pST-P2,then cut by *Aat*II and *Nde*I and inserted into MCS-I of pSS. The *upp* downstream region (980 bp) was amplified using primer pair pST-P3/pST-P4 and cloned between *Sal*I and *Kpn*I sites in MCS-II. After that, *comK* gene was amplified from *B. subtilis* 168 using primer pair pST-P5/pST-P6, and a fragment including *P_ara_* promoter and *araR* gene was also amplified using primer pair pST-P7/pST-P8. C*omK* expression cassette was generated under the control of *P_ara_* promoter by SOE-PCR and inserted between *Bgl*II and *Sal*I sites to yield pST (Figure 2A).

### Generation of *B. subtilis* BUK for Fast Competent Cells Preparation

pST DNA was integrated into *B. subtilis* 168 for constructing the UPRTase deficient host by double-crossover mechanism as previously described [38]. 5FU^R^ recombinants were isolated on MM plate supplemented with 10 µM 5FU, which differed from 20–30 µM 5FU reported by Fabret et al. [19]. Above 10 µM 5FU concentration yielded tiny colonies and did not improve the proportion of *upp*-cassette pop-out. To rule out the single-crossover integration of circular pST combined with spontaneous mutation of *upp,* the physical structure of positive transformants were verified by PCR and DNA sequencing. One colony carrying *comK* cassette at the *upp* locus resulting from the double-crossover integration of linearized pST was designated as *B. subtilis* BUK (Figure 2).

The preparation of competent *B. subtilis* cells and transformation with dsDNA were performed as described previously by Zhang et al. [11] with slightly modifications. Briefly, an overnight LB culture of BUK cells was diluted 10-fold to fresh LB medium. When the cell density reached an OD_600_ (optical density at 600 nm) of 1.0, the culture was supplemented with arabinose at a final concentration of 0.4% (w/v), and continued to be shaken for 2 h. Then, the culture was ready to be transformed. One microlitre PCR product was mixed with 100 µl competent cells and the mixture was incubated at 37°C with shaking at 200 rpm for 90 min to allow the expression of antibiotic resistance gene, and then spread onto LB plates supplemented with appropriate antibiotic.

### Quantitative RT-PCR

Fresh samples of cell cultures were harvested at the exponential phase grown under the same condition in M9 medium to isolated total RNA by using RNAprep pure Kit DP430 (Tiangen, Beijing, China) following the manufacturer’s instructions. RNA samples were then reversed transcribed into cDNA using Quant Reverse Transcriptase with random primers (Tiangen, Beijing, China). The qRT-PCR was carried out by Light Cycler® 480 II (Roche, Basel, Switzerland) with Real Master Mix (SYBR Green) according to the manufacturer’s instructions as follows. In brief, 100 ng of DNA-free total RNA was used in a total reaction volume of 50 µl with 0.25 mM of each primer (Table S1). The fold change values for each gene were normalized to internal control gene *rrn*A and calculated according to the comparative *C*_T_ method [39].

### Confirmation of Mutants by PCR and Sequencing

To confirm the desired mutations, genome DNA was isolated from cells using the TIANamp Bacteria DNA Kit (Tiangen, Beijing, China) following the manufacturer’s instructions. Amplification reactions were carried out using Phusion High-Fidelity DNA Polymerase (New England BioLabs, Ipswich, MA). Mutants were also checked by PCR and DNA sequencing with appropriate primers (Table S1).

## Results

### Construction of Induced Competence of *upp*-defective Strain BUK

It is well known that *B. subtilis* is a naturally competent species, easy to get chromosomal transformation with fully homologous DNA [6]. On the contrary, the recombination frequency for cells double-crossover chromosomal transformation with dsDNA PCR product was relatively low as reported by Fabret et al. [23]. In order to use 5FU for counter-selection and make double-crossover chromosomal transformation with dsDNA PCR product efficiently, a host strain was constructed with *upp* disruption and *comK* cassette insertion. The *comK* cassette was integrated at the *upp* locus on the chromosome seamlessly, which was designated BUK and served as the host strain for markerless mutation delivery. And the extra copy of *comK* enabled BUK to be easily induced into competence under arabinose induction (Figure 2B).

Induced competent cells of BUK could be rapidly prepared and had desired transformation efficiencies of ∼3–5×10^3^ per µg of dsDNA PCR product with arabinose induction at a final concentration of 0.4% (w/v). It was much higher than that of without induction which was hard to obtain transformants. Meanwhile, the maximum of transformation efficiency was several orders of magnitude higher than that by natural transformation induced by starvation as described by Anagnostopoulos et al. [38]. Furthermore, the transformation efficiencies were of ∼1×10^4^ per µg with linearized plasmid DNA, which was similar with the report as described previously by Zhang et al. [11].

### Rationale for the Stimulated Intramolecular Recombination in Mutation Delivery System

**Figure 3:**
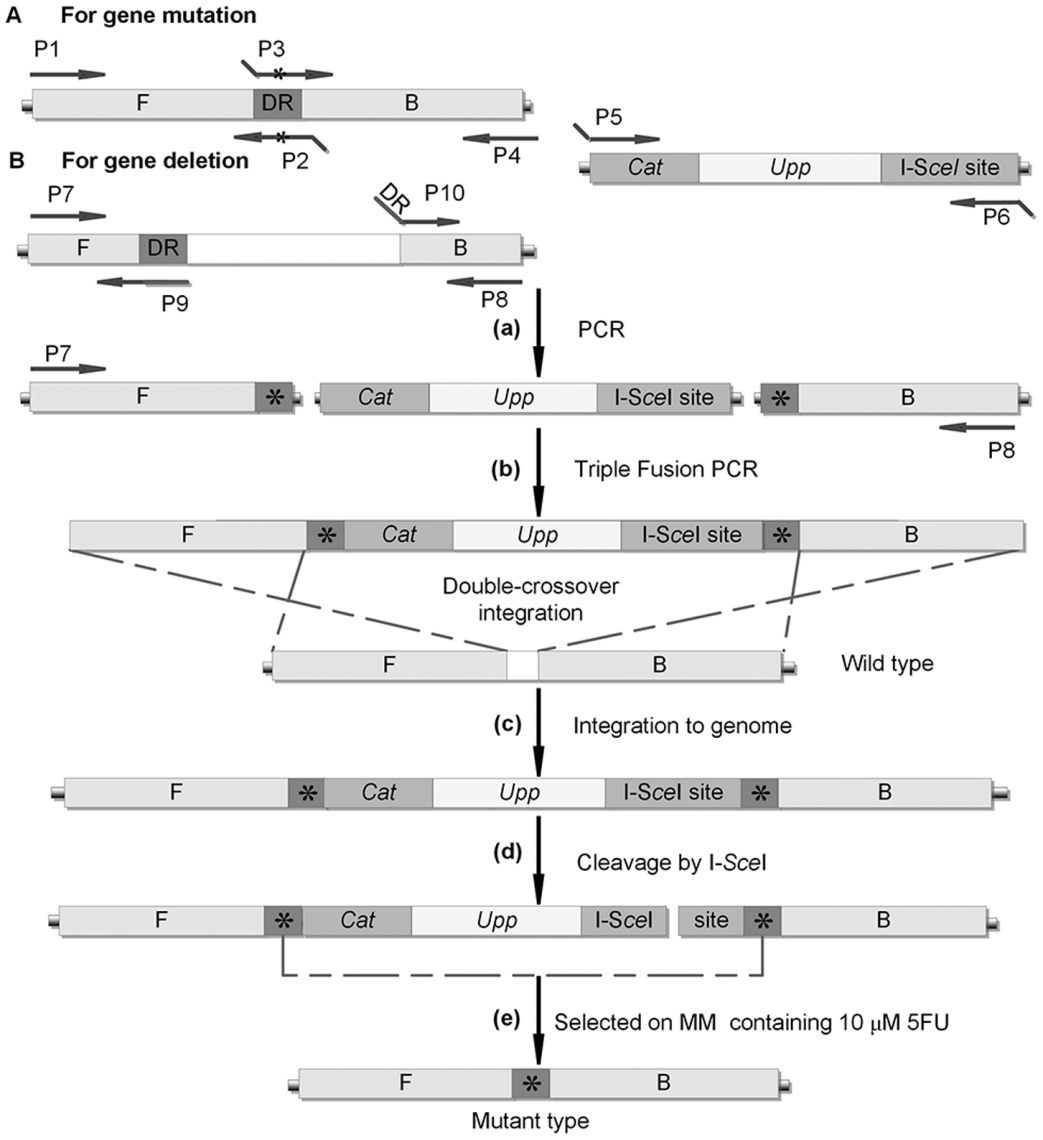
Scheme for mutation delivery procedure in *B. subtilis*. A. (a) For purpose of gene point-mutation, the upstream and downstream fragments carrying the mutation site (*) were amplified by PCR with primer pairs P1/P2 and P3/P4, and *upp*-cassette was amplified by PCR with primer pairs P5/P6, respectively. (b) A triple fusion PCR reaction joined them with the *upp*-cassette. (c) PCR-fused product was used directly to transform BUK, and integration of the *upp*-cassette by first double-crossover event yields 5FU^S^ Cm^R^ transformants. (d) The shuttle vector pEBS-*cop*1 was transformed into the 5FU^S^ Cm^R^ strain to cleavage of the chromosome at the recognition site by I-S*ce*I under xylose induction. (e) The excision of *upp*-cassette through the single-crossover event between the 30 bp DR stimulated by DSB generated a 5FU^R^ Cm^S^ strain that carried only the desired mutation without any other modification in the chromosome. B. For gene deletion, the upstream and downstream fragments carrying the same DR sequence (*) were amplified by PCR with primer pairs P7/P9 and P10/P8. The remainder of the procedure was essentially the same as above described.

For the purpose of obtaining an efficient and precise markerless mutation delivery system, we take advantage of not only efficient genetic recombination during double-crossover chromosomal transformation with dsDNA fragments but also the recombinational repair activity during vegetable growth in *B. subtilis*. The rationale for this system stimulated by DSB is shown in Figure 3. To introduce a point mutation into the chromosome, the upstream and downstream fragments comprising the mutation site are amplified by PCR with appropriate primers, where a 30 bp region of the upstream fragment is identical to the downstream fragment. Then, a triple fusion PCR reaction joins the *upp*-cassette with these two fragments, generating the molecule carrying the *upp*-cassette flanked by 30 bp DR. This dsDNA PCR product is introduced directly into BUK, and aliquots of 10-fold diluted culture are spread on LB plates supplemented with 5 µg/ml chloramphenicol. 5FU^S^ Cm^R^ transformants are isolated where target gene is replaced with the *upp*-tagged mutated copy by a double-crossover homologous recombination event. Then, strains with the expected genotype are selected for eviction of the *upp*-cassette. Vector pEBS-*cop*1 is transformed into the 5FU^S^ Cm^R^ strain on behalf of improvement the pop-out efficiency. Expression of I-S*ce*I endonuclease results in the cleavage of chromosome at the 18 bp recognition site, and cells can survive only by repairing the DSB or by deleting the I-S*ce*I recognition site via intramolecular recombination between DR sequences. Optimum procedure to obtain cells lost *upp*-cassette (5FU^R^ Cm^S^) is described in detail below. A single positive transformant containing pEBS-*cop*1 is incubated overnight in LB medium at 30°C and then diluted 10-fold to fresh LB medium. When the culture is grown to reach an OD_600_ of 1.0, it is supplemented with xylose at a final concentration of 1% (w/v), and continued to be shaken about 6 h for I-S*ce*I endonuclease expression. Aliquots of culture are spread on MM plates containing 10 µM 5FU and incubated for 24 h at 37°C. Excision of *upp*-cassette by the single-crossover event stimulated by DSB repair generates 5FU^R^ Cm^S^ cells. Colonies on 5FU MM plates are picked on LB plates containing 5 µg/ml chloramphenicol and incubated for 12 h at 37°C. Only those 5FU^R^ Cm^S^ colonies are selected for further identification by PCR, and the presence of desired mutation in chromosome is checked by DNA sequencing. Finally, cells can be easily cured of pEBS-*cop1* with heat-sensitive replication by growth at nonpermissive temperatures (50°C) in the absence of the selective antibiotic.

For gene insertion, in-frame deletion, and genome reduction, the 30 bp DR sequence, comprised in the upstream and downstream fragments, is designed as an overlapping region for triple fusion PCR both in the forward primer of the downstream fragment and the backward primer of the *upp*-cassette (Figure 3B), respectively. The remainder of the procedure is essentially the same as above.

### Point-mutagenesis of *ccpN* Gene

**Figure 4:**
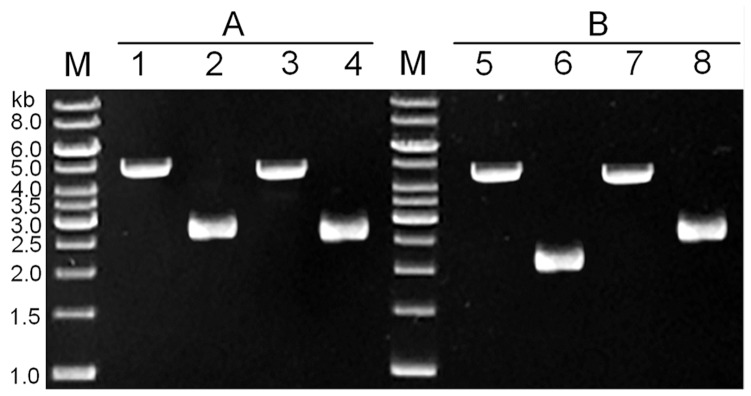
Confirmation of *ccpN* point mutation and *ccpN* in-frame deletion by PCR. The PCR product was analyzed by agarose gel electrophoresis. A 1(Fermentas) was used as a molecular weight marker (lane M). A. Confirmation of the *ccpN* point mutation. Fragments were amplified using ccpN-Mut-P7/ccpN-Mut-P8 as primers. Each lane showed amplified DNA generated from a DNA template: lane 1, BUK-1C (Δ*ccpN*::*upp*-cassette); lane 2, BUK-1 (*ccpN*-mut-Ala 130 to Ser); lane 3, dsDNA PCR fragment (positive control); lane 4, BUK (*ccpN*-wild type) (negative control). B. Confirmation of the *ccpN* in-frame deletion. Fragments were amplified using ccpN-Del-P1/ccpN-Del-P6 as primers. Each lane showed amplified DNA generated from a DNA template: lane 5, BUK-2C (Δ*ccpN*::*upp*-cassette); lane 6, BUK-2 (Δ*ccpN*); lane 7, dsDNA PCR fragment (positive control); lane 8, BUK (*ccpN*-wild type) (negative control).

The transcriptional regulator CcpN mediates CcpA-independent carbon catabolite repression of two gluconeogenic genes *pckA* and *gapB* [40] and controls central carbon fluxes in *B. subtilis*. In order to examine the effectiveness of this strategy for site-directed genetic modification at the native locus, we generated a G-to-T point mutation at position +130 in *ccpN* gene. *Upp*-cassette was PCR-amplified using pSS as template and oligonucleotide pair ccpN-Mut-P3/ccpN-Mut-P4 as primers. 1,379 bp upstream and 1,339 bp downstream fragments of *ccpN* gene were amplified by PCR using two oligonucleotide pairs ccpN-Mut-P1/ccpN-Mut-P2 and ccpN-Mut-P5/ccpN-Mut-P6 as primers, respectively. Both upstream and downstream fragments of *ccpN* gene carried 30 bp DR sequence containing the point mutation G130T. According to the procedure described above, Cm^R^ 5FU^S^ strain was selected after integration of the triple fusion PCR fragment into the chromosome and testified by PCR with primer pair ccpN-Mut-P7/ccpN-Mut-P8 (Figure 4A). Cultures from independently isolated Cm^R^ 5FU^S^ colonies were subjected to 5FU counter-selection, under the xylose induction for I-S*ce*I expression, and the loss of *upp*-cassette was detected by PCR. After the marker-recycling event, the intramolecular homologous recombination between two DR sequences resulted in the desired point mutation. In all the cases, *ccpN* mutant carried solely the expected mutation Ala130Ser (GCC to TCC).

In this case of point-mutagenesis of *ccpN*, a direct correlation between the concentration of xylose and the pop-out efficiency was observed (Table 2). In contrast to the efficiency of 2.8×10^−6^ without xylose induction, the proportion of 5FU^R^ cells stimulated by DSB was between 3×10^−5^ and 8×10^−4^ as the ratio of 5FU^R^ colonies to the viable cells. Moreover, the *upp*-cassette pop-out efficiency in the presence of 1% xylose was about 21-fold higher than that in the presence of 0.5% xylose, and was about 300-fold higher than that of without xylose induction.

**Table 2:** The efficiencies of first double-crossover recombination and counter-selection^a^.

Mutant^b^	DR (bp)	The first double-crossover^c^	xylose induction concentration^d^			
			0%	0.5%	1.0%	2.0%
ccpN*	30	(4.8±0.2)×10^3^	(2.8±0.1)×10^−6^	(3.9±0.4)×10^−5^	(8.4±0.1)×10^−4^	(3.1±0.3)×10^−4^
Δ*ccpN*	30	(3.6±0.3)×10^3^	(2.7±0.4)×10^−6^	(4.0±0.3)×10^−5^	(7.8±0.2)×10^−4^	(4.3±0.3)×10^−4^
ileS*	1691	(4.2±0.2)×10^3^	(7.4±0.4)×10^−6^	(4.3±0.2)×10^−5^	(8.8±0.4)×10^−4^	(4.7±0.5)×10^−4^

a Data are means ± SD from three independent experiments for genetic manipulations.

b ccpN*, point mutation of *ccpN* gene (G130T); ileS*, point mutation of *ileS* gene (G1399A); Δ*ccpN*, in-frame deletion of *ccpN* gene.

c The first double-crossover recombination efficiency was calculated as the number of Cm^R^ colonies/µg of dsDNA PCR product.

d Counter-selection efficiency was calculated as Nr/Nt. Nr, number of 5FU^R^ Cm^S^ colonies in the culture treated by different concentration of xylose; Nt, number of total colonies in culture treated by different concentration of xylose.

### Point-mutagenesis of Essential Gene *ileS*

Essential genes are those indispensable for the survival of an organism under certain conditions. Generally, essential gene is hard to be manipulated because its disruption causes cell death [41]. Here, we try an attempt to deliver a G-to-A point mutation at position +1399 in an essential gene *ileS* coding the isoleucyl-tRNA synthetase. The major experimental process was similar with point-mutagenesis of *ccpN* except for the following minor revision: either upstream or downstream fragment comprised a complete mutant essential gene that kept the cells survival in the first double-crossover recombination event.

The mutagenesis of *ileS* gene was conducted by restriction-ligation method rather than SOE-PCR, since SOE-PCR of several large fragments is not easy to be operated and unwished mutations may be introduced. A 1,733 bp upstream fragment and a 3,093 bp downstream fragment of the *ileS* gene were amplified by PCR using two oligonucleotide pairs ileS-Mut-P1/ileS-Mut-P2 and ileS-Mut-P5/ileS-Mut-P6 as primers, respectively. The downstream fragment comprised a complete mutant essential gene *ileS*. Then, the upstream fragment was cut by *Aat*II and *Xho*I and inserted into MCS-I of pSS. And the downstream fragment was subsequently cloned between *Sal*I and *Bam*HI sites in MCS-II of pSS to yield pSS-*ileS*. The remainder of the procedure is essentially the same as above mentioned in *ccpN* point-mutation. The transformation efficiency was ∼4.2×10^3^ per µg of linearized pSS-*ileS* DNA. After cleavage by I-S*ce*I endonuclease, the pop-out efficiencies of *ileS* point mutation were between ∼4×10^−5^ and ∼9×10^−4^ (Table 2). In addition, the cell growth traits were similar between the parental strain BUK and mutant strain *B. subtilis* BUK-3 in M9 medium, suggesting that *ileS* point-mutation basically did not affected its physiological characteristics.

### In-Frame Deletion of *ccpN* Gene

Here, we generated an in-frame deletion of *ccpN* gene with this system. A 1,110 bp upstream and a 1,140 bp downstream fragments flanked by *ccpN* gene were PCR-generated using two primer pairs ccpN-Del-P1/ccpN-Del-P2 and ccpN-Del-P5/ccpN-Del-P6, respectively. *Upp*-cassette was PCR-amplified using pSS as template and oligonucleotide pair ccpN-Del-P3/ccpN-Del-P4 as primers. Triple fusion PCR product was used to transform BUK. Integration of *upp*-cassette into chromosome inactivated the *ccpN* gene, yielding 5FU^S^ Cm^R^ colonies. Cultures from independently isolated colonies were subjected to 5FU counter-selection, and the proportion of pop-out events was determined by PCR. Mutation process and deletion junctions were testified by PCR with primer pair ccpN-Del-P1/ccpN-Del-P6 and DNA sequencing (Figure 4B). After eviction of *upp*-cassette, the remaining ORF encoded the expected 98-amino-acid peptide that contained 51 N-terminal and 47 C-terminal amino acids of ccpN. The proportion of 5FU^R^ cells was 2.7×10^−6^ without xylose addition. However, the *upp*-cassette pop-out efficiency was still low and needed to be improved. By DSB repair stimulation, the proportion of 5FU^R^ cells raised to between 4×10^−5^ and 8×10^−4^ and that was high enough for fast selection of the desired mutant. The observed highest *upp*-cassette pop-out efficiency was 7.8×10^−4^ at a final xylose concentration of 1% (w/v) (Table 2).

The validity of this method was further tested via investigating the effects of *ccpN* point-mutation and inactivation. In comparison with that of the reference strain BUK, both transcriptional levels of *pckA* and *gapB* were up-regulated in BUK-1 and BUK-2. C*cpN* point-mutation and inactivation seriously affected their physiological characteristics and caused growth defect. Compared with BUK strain, the specific growth rate of BUK-1 decreased from 0.63±0.03 h^−1^ to 0.48±0.01 h^−1^ and the specific growth rate of BUK-2 drastically decreased to 0.28±0.02 h^−1^.

### Deletion of Large-scale Chromosomal Regions

For *B. subtilis*, a large number of potentially dispensable chromosomal loci can be inferred from the complete nucleotide sequence of the chromosome [42]. Fragments such as prophage (-like) regions and the largest operon *pks* (polyketide synthase operon, 1781306–1857233 SubtiList coordinates) are not evolved into regions encoding very indispensable functions, meanwhile, genome minimization affects neither cell viability nor the key physiological [43]. With these considerations in mind, both the 20.5 kb prophage (-like) region (528148–548697 SubtiList coordinates) and the 75.9 kb *pks* operon were chosen as the targets to be deleted, so as to test the availability of marker-free system for genome reduction engineering.

**Figure 5:**
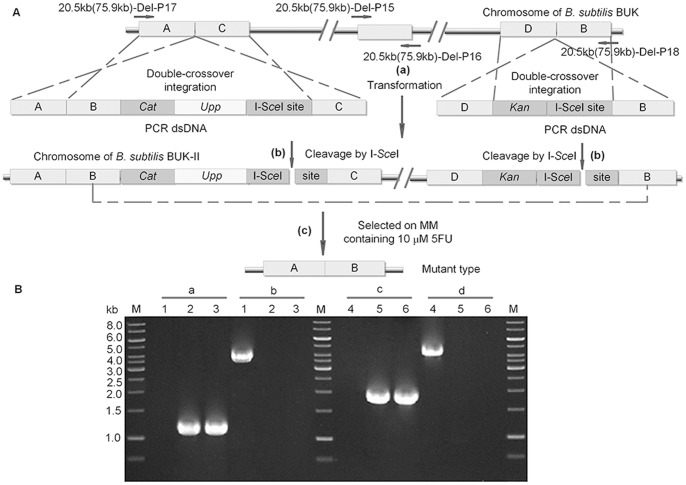
Markerless deletion of large chromosomal regions stimulated by DSB. A. (a) Two dsDNA fragments were generated by fusion PCR and integrated into genome by double-crossover events. (b) The endonuclease I-S*ce*I was expressed from pEBS-*cop*1 under the induction of xylose, and the genome of *B. subtlis* BUK-II was subjected to I-S*ce*I cleavage. (c) Deletion mutants were isolated on MM plate containing 10 µM 5FU and confirmed by PCR. A, B, C, D represent DNA segments selected for homologous recombination. B. Verification of the 20.5 kb and 75.9 kb deletions in BUK by PCR. Fragments were amplified using the primer pairs 20.5 kb-DEL-P15/20.5 kb-DEL-P16 (a), 20.5 kb-DEL-P17/20.5 kb-DEL-P18 (b), 75.9 kb-DEL-P15/75.9 kb-DEL-P16 (c), 75.9 kb-DEL-P17/75.9 kb-DEL-P18 (d). 1 kb DNA Ladder (Fermentas) was used as a molecular weight marker (lane M). (a) Amplification of the *ydcR* gene (534334–535389 SubtiList coordinates) in the 20.5 kb DNA region of *B*. *subtilis*; (b) Amplification of the 20.5 kb DNA fragment of *B. subtilis*; (c–d) Confirmation of the 75.9 kb deletion of BUK was the same as that of 20.5 kb deletion. Amplification of the *pksG* gene (1789763–1791405 SubtiList coordinates) in the 75.9 kb DNA region of *B*. *subtilis* (c) and amplification of the 75.9 kb DNA fragment of *B. subtilis* (d). Each lane showed amplified DNA generated from a DNA template: lane 1, BUKΔ20.5 kb; lane 2, BUK-II (20.5); lane 3, BUK; lane 4, BUKΔ75.9 kb; lane 5, BUK-II (76.9); lane 6, BUK.

We used a new strategy to obtain markerless large-scale deletion mutants (Figure 5). First, we amplified fragment A and fragment B flanking the region to be deleted, fragment C locating at 4–5 kb downstream from the end of fragment A, and fragment D locating at 4–5 kb upstream of fragment B (the length of these fragments is about 1,000 bp, respectively). Fragments A and B were combined to get fragment A-B by SOE-PCR. *Upp*-cassette, fragment A-B and fragment C were fused by triple fusion PCR. Next, 1 µl purified PCR product was used to transform BUK. Transformants were selected on LB plate containing 5 µg/mL chloramphenicol and the positive colony was designated as BUK-I. Another triple fusion PCR product, which contained fragment D, kanamycin resistance gene amplified from pDK and fragment B containing the I-S*ce*I recognition site, was transformed into BUK-I and selected on LB plate containing 5 µg/mL kanamycin. The objective transformant was named BUK-II. For construction of large-scale deletion, pEBS-*cop1* was delivered into BUK-II and cultivated in LB liquid medium at 30°C, with adding 1% xylose at the middle-log growth phase to induce the expression of I-S*ce*I endonuclease. Culture was spread on MM plates containing 10 µM 5FU to obtain colonies in which the *upp*-cassette was excised by intramolecular homologous recombination between two B homologous regions. The chloramphenicol- and kanamycin-sensitive colonies were selected from 5FU MM plate and subjected to further identification by PCR.

After cleavage by I-S*ce*I endonuclease, the pop-out efficiencies of 20.5 kb and 75.9 kb large-scale deletions were between ∼5×10^−6^ and ∼3×10^−5^, which was improved about one to two orders of magnitude compared to that of ∼3×10^−7^ under no xylose addition (Table 3). In addition, for validation the pop-out frequency with one-ended double-strand break, strain BUK-I carrying pEBS-*cop1* was used to generate large-scale deletion. As shown in Table 3, the pop-out frequency with one-ended double-strand break was between 1.5×10^−6^ and 1.5×10^−5^ which was much lower than that of with two-ended double-strand break. Both of these large-scale fragments were individually deleted and no significantly different phenotype was found among the deleted mutants (data not shown).

**Table 3:** Effect of the number of I-S*ce*I sites on the counter-selection for large-scale deletion^a^.

Mutant^b^	DR (bp)	The first double-crossover^c^	Numbers of DSB	xylose induction concentration^d^			
				0%	0.5%	1.0%	2.0%
20.5 kb-DEL	1000	(4.5±0.1)×10^3^	one-ended	(2.8±0.4)×10^−7^	(1.7±0.2)×10^−6^	(4.4±0.1)×10^−6^	(1.5±0.2)×10^−5^
			two-ended	(3.2±0.2)×10^−7^	(5.4±0.1)×10^−6^	(1.4±0.4)×10^−5^	(2.9±0.3)×10^−5^
75.9 kb-DEL	1000	(4.3±0.2)×10^3^	one-ended	(2.5±0.3)×10^−7^	(1.5±0.4)×10^−6^	(4.6±0.1)×10^−6^	(1.1±0.3)×10^−5^
			two-ended	(3.0±0.2)×10^−7^	(6.0±0.3)×10^−6^	(1.0±0.2)×10^−5^	(2.6±0.1)×10^−5^

a Data are means ± SD from three independent experiments for genetic manipulations.

b 20.5 kb-DEL, large deletion of 20.5 kb DNA sequence (528148–548697 SubtiList coordinates) of BUK; 75.9 kb-DEL, large deletion of 75.9 kb DNA sequence (1781306–1857233 SubtiList coordinates) of BUK.

c The first double-crossover recombination efficiency was calculated as the number of Cm^R^ colonies/µg of dsDNA PCR product.

d Counter-selection efficiency was calculated as Nr/Nt. Nr, number of 5FU^R^ Cm^S^ colonies in the culture treated by different concentration of xylose; Nt, number of total colonies in culture treated by different concentration of xylose.

## Discussion

In this study, we report on the development of an easy-to-implement and highly efficient cloning-independent genetic manipulation system in *B. subtilis.* This system exploits *upp* as a counter-selectable marker in concert with DSB stimulation repair caused by I-S*ce*I endonuclease. To the best of our knowledge, it is the first report of establishment of a markerless mutation delivery system in *B. subtilis* stimulated by DSB repair. It bypasses the time-consuming restriction/ligation-dependent vector construction procedure and enhances the pop-out frequency by one to two orders of magnitude after I-S*ce*I cleavage. The DSB-stimulated genetic modification method was successfully used to deliver two point mutations, to realize an in-frame deletion, and to delete two large-scale genomic regions with high efficiency.

A low-frequency of first double-crossover recombinant with dsDNA PCR product is an obstacle for genetic manipulation system. To promote the efficiency of competent cell formation and simplify the preparation process, we generated the host strain BUK by overexpressing *comK* gene encoding the competence transcription factor at the *upp* locus. After a short period of arabinose induction, this engineered strain can be transformed at high efficiency of ∼3–5×10^3^ per µg dsDNA PCR product (Table 2). It is so far the highest genetic recombination efficiency ever reported in *B. subtilis* transformed by double-crossover mechanism with dsDNA PCR product containing heterologous DNA sequences in the central region. In fact, the efficiencies of recombination with PCR fragments vary depending on the intergration sites and the fitness of mutants (data not shown). In addition, the transformation efficiency was of ∼1×10^4^ per µg of linearized plasmid DNA which was consistent with the result of Zhang et al. [11].

In this markerless mutation delivery system, the counter-selection efficiencies were ∼2.8×10^−6^ without xylose addition in point-mutation and in-frame deletion of *ccpN*. In contrast, the pop-out frequency could be enhanced by one to two orders of magnitude under xylose induction. Since the I-S*ce*I cleavage serves not only as a selection tool, but also as a stimulator of the resolution process. From a technical point of view, introduction of insertion, in-frame deletion or point-mutation into the chromosome are equivalent processes. However, construction of large-scale deletion poses an especial issue. Since the frequency of intramolecular recombination depends on the host cell and the physical distance between homologous regions, it is difficult to increase intramolecular recombination efficiency for large deletion of genome. By using the DSB-stimulated replacement method, we deleted two large chromosomal regions of 20.5 kb and 75.9 kb in BUK easily. On the one hand, compared with the high efficiencies of point mutation and in-frame deletion, only between 5×10^−6^ and 3×10^−5^ of the positive transformants survived in large-scale deletions, but all of them were accurate deletion mutants. Since this effect depends on the concentrations of I-S*ce*I in the cell, which can be regulated by different induction concentrations. On the other hand, the one- or two-ended DSB with short patches of homology could be repaired by error-prone single-strand annealing which unwanted mutations might accumulate or by error-free homologous recombination. Indeed, when applying the DSB-stimulated gene replacement method, only one or two DNA lesions are inflicted on the chromosome, thus there are no other hotspots for mutation [31]. According to our experiment results, no increase of mutation rate was observed in cells subjected to DSB-stimulated gene replacement.

This method has been successfully applied to introduce insertions, point mutations and deletions into *B. subtilis* chromosome over thirty various sites in our laboratory. It represents a substantial advance in reiterative genetic manipulation that will save time, effort and expense in functional genomics studies. All these results have proved that this method allows repeated use of the selectable marker for multiple modifications in *B. subtilis* and could be potential applicable in other microorganisms with underdeveloped genetic tools.

## Supporting Information

Table S1 Primers used in this study.
(XLS)
